# Staged Nipple Delay Procedure Expands Candidacy for Nipple-Sparing Mastectomy

**DOI:** 10.1245/s10434-024-16329-y

**Published:** 2024-10-14

**Authors:** Xuanji Wang, Jordan Jackson, Christina Weed, Marissa K. Boyle, Farin F. Amersi, James Mirocha, Armando E. Giuliano, Alice P. Chung

**Affiliations:** 1 Cedars-Sinai Medical Center, Los Angeles, CA USA; 2 Franciscan Breast Surgery at St. Michael, Silverdale, WA USA

**Keywords:** Nipple delay, Nipple-sparing mastectomy, Nipple-areolar complex necrosis, Skin-flap necrosis, Breast cancer, Surgical outcome

## Abstract

**Background:**

Nipple delay (ND) is a staged procedure that improves nipple-areolar complex (NAC) viability in nipple-sparing mastectomy (NSM) patients who are high-risk for NAC or skin-flap necrosis. This study compared postoperative outcomes and risk factors between patients treated with ND-NSM and NSM alone.

**Methods:**

Patient demographics, risk factors for NAC or skin-flap necrosis, tumor characteristics, and operative outcomes were compared between ND-NSM and NSM groups from 2009 to 2023. Univariate and multivariate analyses were performed to identify significant variables associated with NAC or skin-flap necrosis.

**Results:**

Overall, 71 ND-NSM patients and 537 NSM patients were compared. ND-NSM patients had larger breasts (*p* < 0.01), body mass index ≥ 30 (*p* = 0.01), prior breast/chest wall radiation (XRT) [*p* < 0.01], prior breast operations (*p* < 0.01), less axillary surgery (*p* < 0.01), more autologous tissue reconstruction over implant-based reconstruction compared with NSM patients (*p* = 0.02), and more prophylaxis (*p* < 0.01). There were no statistically significant differences between groups in regard to infection, skin-flap necrosis, NAC necrosis, seromas, and hematomas. No patients in the ND-NSM group had NAC necrosis and 1 patient had skin-flap necrosis, compared with 17 and 13 patients in the NSM group, respectively (*p* = 0.24). On univariate analysis, prior XRT was associated with increased risk for skin-flap necrosis (*p* = 0.02). Multivariate analysis showed XRT was associated with skin-flap necrosis (*p* = 0.02) and any necrosis (*p* = 0.01). Breast size was associated with NAC or skin-flap necrosis (*p* = 0.04).

**Conclusion:**

Larger breasts and XRT were risk factors for NAC or skin-flap necrosis; however, despite having more risk factors, ND-NSM patients had very low rates of necrosis. Notably, no nipples were lost. A shared decision should be made with patients regarding the risks and benefits of ND-NSM.

Nipple-sparing mastectomy (NSM) has become an increasingly popular surgical option to enable patients to preserve their nipple-areolar complex (NAC). The NAC is aesthetically an important aspect of the breast, and NAC preservation contributes to patients’ self-image and overall satisfaction after a mastectomy.^1^ NAC preservation is shown to be oncologically safe long term, even in pathogenic mutation carriers.^2–5^

NSM does present with potential risks of positive nipple margin and NAC necrosis. Positive nipple margin warrants resection of the NAC, and NAC necrosis is a complication that is reported in 6–26% of NSM cases.^6–9^ Full-thickness NAC necrosis necessitates surgical debridement, whereas partial NAC necrosis could be managed with meticulous wound care. Risk factors for NAC necrosis include smoking, large and ptotic breast, body mass index (BMI) ≥ 30, prior breast operation, and prior breast/chest wall radiation (XRT).^10,11^

While NAC necrosis is of particular importance in evaluating NSM, skin-flap necrosis also leads to wound-healing complications, scarring, and poor cosmetic outcomes. Few studies have examined the risks of skin-flap necrosis associated with NSM, with a reported incidence of 9–23%.^12,13^ In a series examining 515 NSMs, predictors of skin-flap necrosis include sacrificing the second intercostal perforator, large tissue expander fill volume, and non-lateral inframammary fold incision placement.^13^ Currently, there are even less data as to the risk of skin-flap necrosis in patients with high-risk features undergoing NSM.

First reported by Jensen et al., the nipple delay (ND) is a staged procedure that offers NAC preservation while promoting increased NAC perfusion.^11^ The goal of the ND is to detach the mastectomy skin flaps from the underlying breast tissue to disconnect vasculature coming through the breast tissue to the skin flaps. As a result, ND improves NAC viability.^8,11,14,15^ ND is a particularly useful technique in high-risk NSM patients and has been applied in this patient population, resulting in decreased risk of NAC necrosis.^7,11^ Coupled with a retroareolar biopsy and sentinel lymph node biopsy, when indicated at the time of the first staged procedure, the ND converts high-risk patients to better candidates for NSM. To date, there are few studies that have examined the rate of NAC necrosis in patients undergoing ND. The scarce experiences are small sample studies, and no study has examined the impact of ND on skin-flap necrosis. This study investigates our institutional experience with ND-NSM compared with NSM over the last 15 years. We look to explore the postoperative outcomes and to identify risk factors associated with high-risk skin-flap or NAC necrosis.

## Methods

### Patient Identification

Patients who underwent ND-NSM or NSM alone from 2009 to 2023 were identified from a prospectively maintained institutional database. Operative candidates included patients with cancer diagnoses and high-risk genetic mutation carriers undergoing prophylactic mastectomies. Preoperatively, patients were counseled on the risk of NAC and/or skin-flap partial, treated with conservative management, and full-thickness necrosis, requiring operative debridement. In addition, there is potential need for nipple resection if the retroareolar biopsy was positive for malignancy or carcinoma in situ. The surgeons in our group use the following criteria for recommending an ND procedure: history of breast XRT, current history of smoking, high degree of ptosis, history of periareolar incisions, and large breast size (or breast skin envelope if implants are present). These are patients deemed to be at high risk for skin-flap or NAC necrosis. Patients who did not want to pursue a two-stage ND-NSM were offered skin-sparing mastectomy or NSM, but with accepted higher risk.

### Nipple Delay Technique

Through an inframammary incision, skin flaps are elevated superiorly towards the NAC. The ductal tissue is transected flush at the base of the NAC sharply. The skin flap dissection is carried 2–3 cm superior, medial, and lateral to the NAC. A retroareolar biopsy is obtained at the base of the nipple for histologic examination. Hemostasis is achieved and the inframammary incision is closed without drains or expanders. If indicated, a sentinel lymph node biopsy is completed concurrently. Patients return 7–23 days later for the NSM, reconstruction, and axillary dissection if indicated.

### Outcomes Measures and Statistical Analysis

Patient demographics included age, BMI, mean breast size (weight of breast tissue, in grams), smoking status, diabetes, cardiovascular risk factors, exogeneous steroid use, autoimmune disorder, prior breast operations, prior XRT, neoadjuvant therapy, axillary surgery, type of reconstruction, and operative indication. Of note, breast weight was calculated as combined breast tissue and implant weights for patients with prior breast augmentation. Surgical outcomes of interest included infection, skin-flap necrosis, NAC necrosis, seroma (requiring aspiration), and hematoma (requiring operative evacuation). Notably, infection was documented if patients were treated with antibiotics (oral or intravenous) or were found to have abscesses necessitating aspiration or operative washout. Skin-flap and NAC necrosis was documented if patients had either partial necrosis, treated with conservative management, or full-thickness necrosis, requiring operative debridement.

For numerical variables, an independent samples t-test was used to assess between-group differences, and for categorical variables, the Fisher exact test was used to assess group differences. Logistic regression models were used to assess variables potentially associated with skin-flap necrosis, NAC necrosis, or either skin-flap or NAC necrosis. Multivariable logistic regression models were limited to, at most, three predictors because of the small number of necrosis events. Logistic regression results were reported as odds ratios (OR) and their corresponding 95% confidence intervals (CI). The two-sided 0.05 significance level was used throughout. Statistical calculations were performed using SAS version 9.4 (SAS Institute, Cary, NC, USA).

## RESULTS

### Patient Factors and Postoperative Outcomes

Seventy-one patients underwent ND-NSM and 537 patients received NSM alone. There were no significant differences between groups with regard to age, diabetes, cardiovascular risk factors, exogeneous steroid use, autoimmune disorder, and smoking status. The ND-NSM group had larger breasts by mean weight (500 g [120–1094 g] vs. 378 g [48–1528 g]; *p* < 0.01), was more likely to have BMI ≥ 30 (16.9% vs. 7.4%; *p* = 0.01), had a higher rate of prior XRT (12.7% vs. 3.5%; *p* < 0.01), and had a higher rate of prior breast operations (52.1% vs. 23.5%; *p* < 0.01). Compared with NSM, the ND-NSM cohort was less likely to undergo axillary surgery (sentinel lymph node biopsy or axillary dissection) (64.8% vs. 95.3%; *p* < 0.01), was more likely to have autologous tissue reconstruction over implant-based reconstruction (15.5% vs. 6.7%; *p* = 0.02), and was more likely to undergo prophylactic operation (14% vs. 0.4%; *p* < 0.01) [Table 1].

**Table 1:** Clinical features of nipple delay and nipple sparing mastectomy

	ND-NSM	NSM	*p*
	Patients (*n*=71)	Patients (*n*=537)	
Demographics			
Age (yr)	48.8	48.8	0.9
Breast size (g)	500	378	< 0.01*
Body mass index, *n* (%)			0.01*
≥30	12 (16.9)	40 (7.4)	
<30	59 (83.1)	497 (92.6)	
Smoking, *n* (%)			0.76
yes	2 (2.8)	24 (4.5)	
no	69 (97.2]	513 (95.5)	
Diabetes, *n* (94)			
yes	2 (2.8)	19 (3.5)	0.99
no	69 (97.2)	518 (96.5)	
Cardiovascular risk factors, *n* (%)			
yes	1 U.41	19 (3.5)	0.50
no	70 (98.6)	518 (96.5)	
Exogenous steroid use			
yes	0(0)	5 (0.9)	> 0.99
no	71 (100)	532 (99.1)	
Autoimmune disorder, *n* (%)			
yes	7 (9.9)	25 (4.7)	0.08
no	64 (90.1)	512 (95.3)	
History of Breast/chest radiation, *n* (%)			< 0.01*
yes	9 (12.7)	19 (3.5)	
no	62 (87.3)	518 (96.5)	
Prior breast surgery, *n* (%)			< 0.01*
None	34 (47.9)	411 (76.5)	< 0.01*
Augmentation	10 (14.1)	32 (6)	0.02*
Reduction/mastopexy	8 111.3)	2 10.4)	< 0.01*
Excision/segmental mastectomy	19 (26.8)	92 (17.1)	C.C7
Neoadjuvant therapy, *n* (%)			> 0.99
Yes	13 (18.3)	102 (19.0)	
No	58 (81.7)	435 (81.0)	
Axillary surgery, *n* (%)			< 0.01*
None	25 (35.2)	25 (4.7)	< 0.01*
SLNB	39 (54.9)	392 (73.0)	< 0.01*
SLNB + ALND	7 (9.9)	120 (22.3)	
Type of reconstruction, *n* (%)			0.01*
Tissue expander	53 (74.6)	434 (80.8)	0.27
Direct to implant	4 (5.6)	61 (11.4)	0.22
Autologous tissue	11 (15.5)	36 (6.7)	0.02*
Unknown/no Reconstruction	3 (4.2)	6 (1.1)	–
Operative indication, *n* (%)			< 0.01*
Cancer	61 (86)	535 (99.6)	
Prophylaxis	10 (14)	2 (0.4)	

Analysis donewith t-test and Fisher’s exact test | p values with * are significant

For 90-day postoperative complications, there were no statistically significant differences between groups with respect to infection, skin-flap necrosis, NAC necrosis, seroma, and hematoma. Notably, there were no patients with NAC necrosis in the ND-NSM group, versus 17 (3.1%) patients in the NSM group (*p* = 0.24). Four of the patients with full-thickness NAC necrosis in the NSM group required operative NAC resection. Only 1 (1.4%) patient had inferior full-thickness skin-flap necrosis necessitating operative skin resection in the ND-NSM cohort compared with 13 (2.4%) patients in the NSM cohort (*p* = 1.0) [Table 2].

**Table 2:** Postoperative outcomes of nipple delay and nipple-sparing mastectomy

	ND-NSM [*n* = 71]	NSM [*n* = 537]	*p*-Value
90-day postoperative complications			
Complications [*n* (%)]			
None	59 (83.1)	437 (79.0)	0.53
Infection	7 (9.9)	62 (11.2)	0.84
Skin-flap necrosis	1 (1.4)	13 (2.4)	>0.99
Nipple-areolar complex necrosis	0 (0)	17 (3.1)	0.24
Seroma [requiring aspiration]	1 (1.4)	15 (2.7)	>0.99
Hematoma [requiring evacuation]	3 (4.2)	9 (1.6)	0.15

Analysis was performed using the t-test and Fisher’s exact test

*ND* nipple delay, *NSM* nipple-sparing mastectomy

### Univariable and Multivariable Risk Factors

Univariable analysis was performed on factors considered as potentially related to skin-flap or NAC necrosis. These factors included age, smoking, BMI, diabetes, ND, prior XRT, prior breast operations, axillary surgery, autologous reconstruction, and neoadjuvant therapy. Only prior XRT was associated with a significant risk for skin-flap necrosis (*p* = 0.02).

Multivariate logistic regression was performed to determine whether there was any association between breast size, history of XRT, and prior breast surgery with skin-flap necrosis, NAC necrosis, and NAC or skin-flap necrosis. Prior XRT was associated with increased risk of skin-flap necrosis (OR 7.15, 95% CI 1.31–39.01; *p* = 0.02) [Table 3], however no factors were significantly associated with increased risk of NAC necrosis (Table 4). Breast size (OR 1.17, 95% CI 1.01–1.35; *p* = 0.04) and XRT (OR 5.29, 95% CI 1.51–18.53; *p* = 0.01) were associated with increased risk for NAC or skin-flap necrosis (Table 5).

**Table 3:** Multivariable analysis: risk factors for skin-flap necrosis

	OR	95% CI	*p* Value
Breast size, g	1.14	0.91–1.42	0.26
History of breast/chest radiation [*n* (%)]			
No	Reference		
Yes	7.15	1.31–39.01	0.02*
Prior breast surgery [*n* (%)]			
No	Reference		
Yes	0.92	0.22–3.87	0.91

*OR* odds ratio, *CI* confidence interval

*Indicates statistical significance

**Table 4:** Multivariable analysis: risk factors for NAC necrosis

	OR	95% CI	*p* Value
Breast size, g	1.17	0.98–1.41	0.08
History of breast/chest radiation [*n* (%)]			
No	Reference		
Yes	3.23	0.55–18.95	0.19
Prior breast surgery [*n* (%)]			
No	Reference		
Yes	0.85	0.25–2.91	0.80

*OR* odds ratio, *CI* confidence interval, *NAC* nipple-areolar complex

**Table 5:** Multivariable analysis: risk factors for NAC or skin-flap necrosis

	OR	95% CI	*p* Value
Breast size, g	1.17	1.01–1.35	0.04*
History of breast/chest radiation [*n* (%)]			
No	Reference		
Yes	5.29	1.51–18.53	0.01*
Prior breast surgery [*n* (%)]			
No	Reference		
Yes	0.88	0.34–2.26	0.78

*OR* odds ratio, *CI* confidence interval, *NAC* nipple-areolar complex

*Indicates statistical significance

## Discussion

ND preserves the aesthetic advantages of NSM in patients at high-risk for NAC or skin-flap necrosis. Since ND was first introduced at our institution, it has gained popularity and has been safely executed for the last 15 years. Review of our experience shows that ND-NSM is performed more often in patients with larger breasts, BMI ≥ 30, prior XRT, prior breast operations, and for prophylaxis, compared with NSM patients. Although the ND-NSM patients had higher-risk for NAC and skin- flap necrosis, the results showed a lower incidence of NAC and skin-flap necrosis compared with the NSM group. Most importantly, there was no nipple loss in the ND-NSM cohort.

Our study also identified that ND-NSM patients were less likely to undergo axillary surgery (including sentinel lymph node biopsy and axillary dissection). This is because a larger portion of the ND-NSM patients were high-risk mutation carriers undergoing prophylactic mastectomies. Lee et al. examined the time to definitive treatment in patients with breast cancer and showed that the difference between ND-NSM and NSM patients at their institution was only 16 days.^16^ Other studies also showed no significant delay in breast cancer treatment.^11,15^ At our institution, the average time between the ND and NSM was similar. In addition, ND-NSM patients were more likely to receive autologous reconstruction than NSM patients. Other studies in the literature have varying percentages of autologous reconstruction versus tissue expander placement versus direct to implant.^11,14,17^ These differences in reconstructive patterns are likely driven by provider practice preferences and patients’ choice.

Some evidence suggests that autologous tissue reconstruction preserves dermal plexus perfusion, and in turn promotes NAC and skin-flap survival.^18^ Bertoni et al. reported that patients who underwent NSM with autologous reconstruction had less NAC ischemic complications, including epidermolysis, partial necrosis, and full-thickness necrosis requiring NAC removal, compared with patients with immediate implant-based reconstruction.^17^ Furthermore, Lee et al. reviewed skin-sparing mastectomy patients undergoing autologous tissue reconstruction and expander-based reconstruction,^19^ and showed that the mastectomy skin-flap necrosis rate was significantly lower in the autologous group. While our ND-NSM cohort had a higher rate of autologous reconstruction compared with the NSM cohort, the univariate analysis showed that the type of reconstruction was not associated with an increased risk of NAC or skin-flap necrosis.

Other studies have evaluated the effect of ND-NSM on NAC necrosis; few, if any, have examined the impact of ND-NSM on skin-flap necrosis. It is important to include skin-flap necrosis when assessing operative outcomes, as it may lead to reoperation, wound care complications, risk of implant loss, and delay to adjuvant therapy. Furthermore, our robust experience speaks to the applicability of ND-NSM over time as an option for patients who may not be optimal candidates for NSM. It is important to note that we specifically assessed full-thickness skin-flap and NAC necrosis necessitating operative debridement. Partial-thickness necrosis was treated with nitroglycerin paste, silvadene, antibiotics, hyperbaric oxygen, and other local wound care.

The major disadvantage of the ND-NSM is that it is a two-stage procedure. The surgeons at our institution offer this procedure to patients at high risk for skin flap or NAC necrosis. Those who are not willing to have a two-stage procedure may opt for skin-sparing mastectomy without preservation of the NAC, or may choose to proceed with NSM while accepting a higher risk of necrosis. In counseling the patients, we discuss the rationale for the ND-NSM, and it is ultimately a shared decision. In our experience, patients who are motivated to preserve their nipples are not deterred by a second operation. Another disadvantage of ND-NSM is the costs associated with additional operations. This must be balanced with the potential risk for NAC or skin-flap necrosis that leads to return to the operating room, increased outpatient visits, infections requiring prolonged antibiotics, reconstruction revisions, and the emotional tax on patients. The first stage of the ND-NSM is an outpatient procedure and well tolerated. The tissue from subareolar biopsy is only examined on final surgical pathology to minimize the false negative rates of frozen section. Finally, a staged approach enables a more informed discussion with the patients in cases necessitating NAC resection or axillary dissection. A systematic cost analysis is necessary to delineate the cost trade-offs.

A limitation of this study is that this was a single-center retrospective review. Patients were stratified as ND-NSM candidates based on provider-patient discussions since the risk factors for skin-flap and NAC necrosis are not absolute contraindications for NSM. Ultimately, these high-risk patients are identified by providers and the lack of randomization to the study weakens the strength of the results. This study would be strengthened by a prospective patient-reported outcome study to assess patient satisfaction with ND-NSM compared with NSM.

## Conclusion

Nipple preservation, when oncologically safe, should be offered for patients as the nipple plays a large role in long-term patient satisfaction and psychological well-being.^20^ The incidence of NAC necrosis after NSM is reported at 6–26% in the literature, while at our institution the NAC necrosis rate is only 3.1%, and 0% after ND. Furthermore, Ito et al. showed that breasts more than 400 g are associated with NAC necrosis 35% of the time, while the mean weight in the ND-NSM cohort was 500 g.^21^ Since adoption of the ND, our NAC necrosis rate has been 0% and only one patient had skin-flap necrosis. ND requires detailed patient education to the goal of the staged procedure as well as the possibility of needing NAC resection. In our experience, patients are motivated to preserve their nipple when able. Therefore, a shared decision should be made with patients based on the risks and benefits of the ND-NSM.
